# Statistical Distribution and Entropy of Multi-Scale Returns: A Coarse-Grained Analysis and Evidence for a New Stylized Fact

**DOI:** 10.3390/e28020172

**Published:** 2026-02-02

**Authors:** Alejandro Raúl Hernández-Montoya

**Affiliations:** 1 Instituto de Investigaciones en Inteligencia Artificial, Universidad Veracruzana, Campus Sur, Calle Paseo No 112, Lote 2, Colonia Nueva Xalapa, Xalapa 91097, Veracruz, Mexico; alhernandez@uv.mx; 2 Facultad de Física, Universidad Veracruzana, Campus Sur, Calle Paseo No 112, Lote 2, Colonia Nueva Xalapa, Xalapa 91097, Veracruz, Mexico

**Keywords:** price runs, market efficiency, multi-scale returns properties, empirical analysis

## Abstract

Financial time series often show periods during which market index values or asset prices increase or decrease monotonically. These events are known as price runs, uninterrupted trends, or simply runs. By identifying such runs in the daily DJIA and IPC indices from 2 January 1990 to 17 October 2025, we construct their associated returns to obtain a non-arbitrary sample of multi-scale returns, which we call trend returns (TReturns). The timescale of each multi-scale return is determined by the exponentially distributed duration of its corresponding run. We empirically show that the distribution of these coarse-grained returns exhibits distinctive statistical properties: the central region displays an exponential decay, likely resulting from the exponential distribution of trend durations, while the tails follow a power-law decay. This combination of exponential central behavior and asymptotic power-law decay has also been observed in other complex systems, and our findings provide additional evidence of its natural emergence. We also explore the informational properties of multi-scale returns using three measures: Shannon entropy, permutation entropy, and compression-based complexity. We find that Shannon entropy increases with coarse-graining, indicating a wider range of values; permutation entropy drops sharply, revealing underlying temporal patterns; and compression ratios improve, reflecting suppressed randomness. Overall, these findings suggest that constructing TReturns filters out microscopic noise, reveals structured temporal patterns, and provides a complementary and clear view of market behavior.

## 1. Introduction

Since the late eighteenth century, the success of the physical sciences inspired economists, especially within the neoclassical tradition, to construct economic theory following the methodological structure of physics, and in particular analytical mechanics [1]. In this sense, physics and mathematics have long played a central role in shaping the development of the economic sciences.

Since the mid-1990s, challenging problems arising in economic and social systems have attracted growing interest from the physics community. This effort gave rise to econophysics, a field in which techniques from computational statistical mechanics are applied to economic systems from both theoretical and empirical perspectives. Over time, researchers from computer science, biology, big data, artificial intelligence, and related fields joined this movement, approaching economic phenomena with both applied and foundational goals. The broader research area that studies systems composed of large numbers of particles or agents using interdisciplinary tools is now commonly referred to as the field of *Complexity Sciences*.

Due to their nature, economic and social systems cannot be easily manipulated through controlled experiments. Nevertheless, the massive growth of computational power, together with the availability of large datasets accumulated over decades, and in some cases centuries, opened the door to an empirical and quasi-experimental approach to socio-economic phenomena. Rich data sources now include financial prices, transaction records, personal income archives, large-scale surveys, and, more recently, vast datasets extracted from social networks that capture information on human interactions, preferences, opinions, and political behavior.

In this paper, following an empirical methodology rooted in econophysics, we analyze the financial time series of two market indices: the American Dow Jones Industrial Average (DJIA) and the Mexican Stock Exchange Index (IPC), described in detail in Section 4.

### 1.1. Stylized Facts

From this empirical standpoint, several non-trivial and remarkably universal statistical properties of financial time series, known as *stylized facts*, have been documented. Although often formulated in qualitative terms, these properties impose rigorous constraints on any statistical description, and reproducing all of them simultaneously remains very challenging for standard stochastic models [2]. To the best of our knowledge, no existing theory or model is currently able to explain the origin of all stylized facts simultaneously. However, agent-based modeling has been suggested as a promising methodological approach to address this limitation [3,4,5]. This view is reinforced by recent developments in kinetic and microstructural modeling [6,7], as well as by the broader econophysics literature, where stylized facts are recognized as signatures of complex-system behavior [8].

Interestingly, stylized facts appear not only in financial markets but also in other complex dynamical systems, sometimes surprisingly simple ones, such as Conway’s Game of Life [9].

We summarize below some of the most relevant stylized facts [2]:

Heavy tails: Return distributions are leptokurtic and exhibit asymptotic power-law decay.
Absence of linear autocorrelations: Autocorrelations of returns are negligible except at very short time scales, depending on the market and sampling frequency.
Gain–loss asymmetry: Large downward movements in stock prices or indices are more frequent than equally large upward movements.
Volatility clustering: Periods of high volatility tend to cluster in time.
Other: Additional properties include aggregational Gaussianity, volume-volatility correlations, long memory in the autocorrelation of absolute returns, and many others.

Dozens of statistical properties have now been identified as stylized facts. Their study is essential for testing and validating numerical and agent-based models of financial systems, and they remain an active area of research for the academic communities in finance and econophysics [4,5,8].

### 1.2. Coexistence of Exponential and Power-Law Distributions Across Diverse Complex Systems

Although the concept of stylized facts is primarily associated with finance, several statistical properties appear not only in financial markets but across a wide range of complex systems. A recurring statistical pattern in such systems is the coexistence of an exponential central region and asymptotic power-law tails. This pattern appears in settings with radically different microscopic rules and physical substrates: the distribution of wealth and income follows an exponential law in the low and central regions, with heavy-tailed extremes in all societies [10]; cultural ranking processes, such as the Dutch Top 2000 pop-song list [11]; cellular automata, such as “the Game of Life”, generate thermal and superthermal wealth-like distributions [12]; suprathermal particle populations in space and atmospheric plasma  [13,14,15]; hydrodynamic extensions of hadronic production models in heavy-ion collisions [16]; and the autocorrelation envelopes of multi-scale financial returns [17], and others complex systems. This recurrence of the same statistical form across socioeconomic systems, astrophysical plasmas, cellular automata, cultural dynamics, and high-energy physics suggests the existence of a common organizing principle giving rise to the observed statistical patterns. Although a unified theory explaining why this combination arises still remains to be developed, the empirical regularity is robust.

We mention these examples because, in this paper, we add another instance of the joint emergence of these two distributions in a complex system. The stochastic process introduced here, multi-scale returns, is itself governed by the same statistical structure: exponential decay in the central region and asymptotic power-law in the tails, in close analogy with the systems mentioned above.

## 2. Data Sample

The data analyzed in this work consist of the daily closing values of two financial indices: the American Dow Jones Industrial Average (DJIA) and the Mexican Price and Quotations Index (IPC, Índice de Precios y Cotizaciones), covering the period from  2 January 1990 to 17 October 2025.

The IPC series was initially obtained from historical datasets provided to us by the Bolsa Mexicana de Valores (BMV) for academic use, as well as from datasets that were publicly accessible at the time from the Bank of Mexico. These historical records were later complemented with additional public sources to construct a continuous and internally consistent IPC dataset.

For the DJIA series, publicly accessible historical records were used, including datasets available at the time from the Central Bank of Brazil. The data were publicly accessible when retrieved by the authors, as they were originally downloaded from diverse public portals, some of which subsequently withdrew their corresponding historical files. Both the IPC and DJIA series required further preprocessing to address common issues found in long financial time series, such as repeated dates, duplicated rows, inconsistent timestamps, and entries for non-trading days. After the full cleaning and reconstruction procedure, the IPC and DJIA datasets consisted of 8996 and 9011 daily observations, respectively.

The final reconstructed datasets used in this study, together with all derived observables analyzed in the following sections, are publicly available at the companion Zenodo repository: https://doi.org/10.5281/zenodo.17781871.

Below, we describe how the observables analyzed in this work are constructed from the two datasets.

## 3. Methodology and Construction of the Multi-Scale Returns

**Figure 1:**
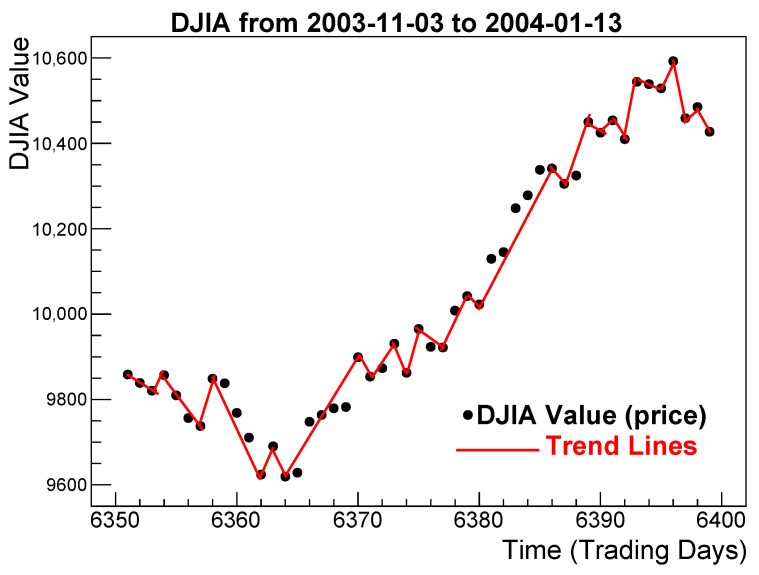
Uninterrupted trends in the DJIA during the period 3 November 2003 to 13 January 2004. Red segments connect the starting and ending points of each trend. Shown for illustration.

The evolution of an asset price on a time-series chart often reveals periods during which the price (or index value) increases or decreases monotonically. We refer to these periods as runs, uninterrupted price trends, or simply trends. These alternating upward and downward price movements form the foundation of our construction of multi-scale returns. For illustration, Figure 1 shows examples of uninterrupted trends in the DJIA during the period 3 November 2003–13 January 2004.

More formally, let ${\left\{P_{t}\right\}}_{t=1}^{N}$ denote the time series of daily closing prices (or index values) of a financial asset. A standard quantity used to study price variations is the log-return, defined for a sampling interval, or time scale Δt as

(1) $$R\left(t,\Delta t\right)=\log P_{t+\Delta t}-\log P_{t}.$$

In this work, we analyze sequences of log-returns with variable time scale Δt, determined endogenously from the data and corresponding precisely to the durations of uninterrupted monotonic price trends, or price runs. Returns constructed in this way are called multi-scale returns, trend returns or simply TReturns.

### 3.1. Uninterrupted Trends

Given a financial time series ${\left\{P_{t}\right\}}_{t=1}^{N}$, an uninterrupted trend is a subsequence of prices in which the values move monotonically in a single direction, either upward or downward, for $m_{i}$ consecutive days. More formally, for a given starting time $l_{i}$, an uninterrupted trend of duration $m_{i}$ consists of the $m_{i}+1$ consecutive prices up to the first change in direction

$$P_{l_{i}},P_{l_{i}+1},\ldots ,P_{l_{i}+m_{i}}.$$

The trend is classified according to its monotonicity as

- Uptrend:
  $$P_{l_{i}}<P_{l_{i}+1}<\cdots <P_{l_{i}+m_{i}};$$
- Downtrend:
  $$P_{l_{i}}>P_{l_{i}+1}>\cdots >P_{l_{i}+m_{i}}.$$

The duration $m_{i}$ (measured in trading days) represents the number of steps during which prices continue moving monotonically before reversing direction. By construction, the ending point of one trend is the starting point of the next. It follows that each uptrend is followed by a downtrend and vice versa; in other words, uptrends and downtrends alternate.

This process partitions the entire price series into *K* uninterrupted trends whose durations satisfy

$$\sum_{i=1}^{K}m_{i}=N-1.$$

It is well known that the duration of uninterrupted monotonic trends (or runs) decays exponentially. This result is based on classical probability theory: runs arising in symmetric discrete random walks with unit step size have geometrically distributed lengths [18], whose continuous analogue is the exponential distribution. Additional empirical evidence of exponential decay in financial runs has been reported in [19]. Our previous work confirms this behavior for stock index data: trend length distributions are geometric, with parameters close to 1/2 for more mature markets, and thus become exponential in the continuous limit [20].

The key feature of multi-scale returns is that they are not computed over fixed time intervals, but over the duration of each run. Since the empirical distribution of trend durations is exponential, the multi-scale return series is constructed over an exponentially distributed set of time scales. Consequently, an exponential regime is expected to propagate naturally into the multi-scale return distribution, specifically into its low and medium value region, a result that we demonstrate empirically in this paper (see Section 4.2).

### 3.2. Definition of Multi-Scale (Trend) Returns

For each uninterrupted trend starting at discrete time $l_{i}$ with duration of $m_{i}$ days, we define the associated multi-scale return, or trend return (TReturn), as the logarithmic return calculated between the initial and final prices of the trend:

(2) $$R_{i,m_{i}}:=\log P_{l_{i}+m_{i}}-\log P_{l_{i}}=\log\left(\frac{P_{l_{i}+m_{i}}}{P_{l_{i}}}\right).$$

Thus, each multi-scale return $R_{i,m_{i}}$ summarizes the cumulative price variation over the full duration of trend *i*, whether upward or downward.

Collecting all uninterrupted trends in the price series yields the sequence

$$R_{1,m_{1}},\;R_{2,m_{2}},\;\ldots ,\;R_{K,m_{K}},$$

which we refer to as the multi-scale return series.

Although TReturns are formally defined as the logarithmic change between the initial and final values of each uninterrupted monotonic trend, in practice they are more efficiently computed by aggregating daily log-returns until a sign change is detected.

## 4. Data Analysis

This section characterizes the run durations for both indices (DJIA and IPC). First, we confirm the well known statistical result that run durations decay exponentially by fitting their empirical distributions. We then analyze the multi-scale returns constructed from these exponentially distributed run durations for both markets and show, through the corresponding fits, that the multi-scale return distributions associated with these indices are well described by a combination of exponential behavior in the central region, covering both positive and negative values, and power-law decay in the tails. All data analysis is performed using the ROOT framework [21].

### 4.1. Descriptive Statistics

Table 1 summarizes the descriptive statistics of the run durations. For both indices, run lengths are short, typically averaging two to three trading days, and exhibit positive skewness and high leptokurtosis, reflecting the presence of a small number of unusually long trends.

### 4.2. Exponential Decay of Run-Duration Distributions

All subsequent exponential fits were performed using the same ROOT-based procedure described here.

As noted at the end of Section 3.1, it is well known that the distribution of run lengths is accurately described by an exponential decay (with the exception of occasional outliers), of the form

(3) y(ℓ)=exp(C−γℓ),

where *C* and γ are parameters to be estimated empirically.

The exponential fit is performed by adjusting a straight line to the base-10 logarithm of the histogram. If N(ℓ) denotes the number of runs (occurrences) of length *ℓ*, ROOT fits the relation

(4) $$\log_{10}N\left(\ell \right)=a+b\;\ell ,$$

where the parameters *a* and *b* are reported by ROOT as Constant and Slope, respectively. The corresponding exponential model implied by Equation (4) is

(5) $$N\left(\ell \right)\propto 10^{a}\;10^{\;b\ell }=\exp\left(a\ln10+b\;\ell \;\ln10\right).$$

By comparing this expression with the standard exponential decay defined by  Equation (3), one directly obtains

(6) C=aln10,γ=−bln10.

**Figure 2:**
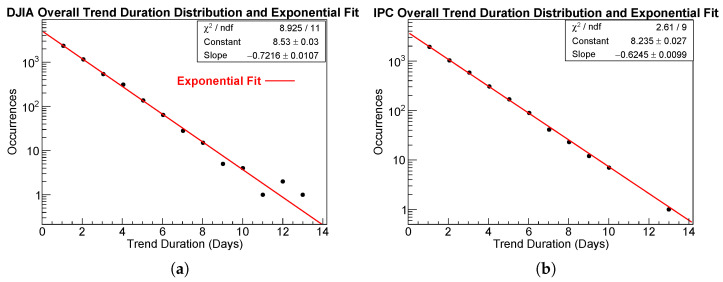
Log-linear distributions of uninterrupted trend duration in days for the (**a**) DJIA and (**b**) IPC. The empirical distributions (black bullets) are well described by the exponential model (red lines). The corresponding fit parameters are displayed in the Figure and also reported in Table 2.

For consistency with the ROOT output and to avoid unnecessary reparameterization, all numerical results and figures reported in this section display the original ROOT parameters *a* (Constant) and *b* (Slope). Exponential fits of the run duration distributions for the two markets are shown in panels of Figure 2a,b, and the corresponding fit parameters for the overall trend distributions are reported in Table 2. Both markets display a clear exponential decay.

#### Goodness of the Exponential Fit: Overall Run Duration Distribution

The exponential fits show a satisfactory goodness of fit in both cases. The $\chi^{2}/\mathrm{ndf}$ values fall within the acceptable range for a single parameter exponential model, and the log-linear plots show no curvature or systematic deviations. In particular, the DJIA fit yields $\chi^{2}/\mathrm{ndf}$ = 8.93/11, while the IPC fit yields $\chi^{2}/\mathrm{ndf}$ = 2.61/9. These values, together with the exponential fits shown in Figure 2, confirm that trend durations are exponentially distributed, and that trend termination follows an approximately constant hazard rate.

### 4.3. Runs, Uptrends, and Downtrends: Separate Exponential Fits

A more detailed picture emerges when the duration distributions of uptrends and downtrends are analyzed separately. Table 3 and Table 4 present the descriptive statistics and exponential fit parameters for each case.

**Figure 3:**
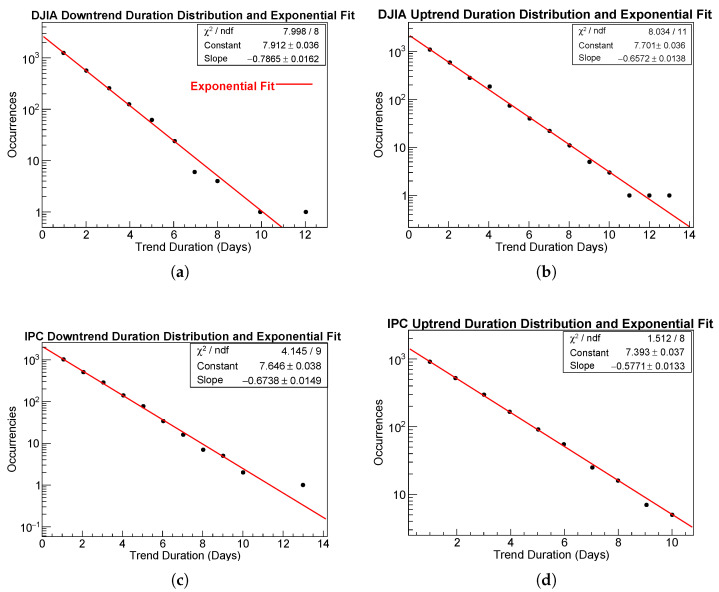
Log-linear distributions of uninterrupted trends duration (in days) for the following cases: (**a**) DJIA downtrends, (**b**) DJIA uptrends, (**c**) IPC downtrends, and (**d**) IPC uptrends. In all cases, the exponential model (red line) provides a good description of the data (black bullets). Fit parameters are shown in the Figure and summarized in Table 4.

Figure 3 shows the four exponential fits for uptrend and downtrend durations in the DJIA and IPC markets. The exponential model provides a good description of all four samples of trend durations.

#### Goodness of the Exponential Fit: Separate Upward and Downward Runs Duration Distributions

The goodness of the exponential fits is again satisfactory for both indices. The $\chi^{2}/\mathrm{ndf}$ values reported in Table 4 indicate that the exponential model captures the empirical distributions of upward and downward run durations with no statistically significant deviations. Visual inspection of the four plots in Figure 3a–d confirms the absence of systematic departures from exponential decay: all points fall on straight lines across the full range of observed durations, with the only possible exception of the longest-duration point for DJIA and IPC downtrends. Overall, the exponential decay is preserved in all four cases. Several features are worth highlighting:

- Downtrends decay faster than uptrends in both markets. For the DJIA, the slope changes from b=−0.657 (uptrends) to b=−0.787 (downtrends). For the IPC, a similar pattern is observed, with b=−0.577 vs. b=−0.674. The difference is moderate but systematic across both indices, suggesting a weak but consistent directional asymmetry in trend dynamics.
- With the exception of IPC uptrends, the $\chi^{2}/\mathrm{ndf}$ values range from approximately 0.5 to 1.0, indicating excellent fits with no systematic patterns in the residuals. For IPC uptrends, the $\chi^{2}/\mathrm{ndf}$ = 1.512/8 also indicates a very good fit. This relatively low value suggests that the exponential model closely matches the empirical distribution, possibly reflecting conservative ROOT error estimates or small statistical fluctuations in the data.
- IPC uptrends exhibit the smallest kurtosis (3.209), indicating a relatively lighter tail compared to the other cases, whereas DJIA uptrends display the heaviest tail (kurtosis ≈5.86).

Overall, the exponential law remains robust across all subsets of runs.

### 4.4. TReturns Distribution Analysis

**Figure 4:**
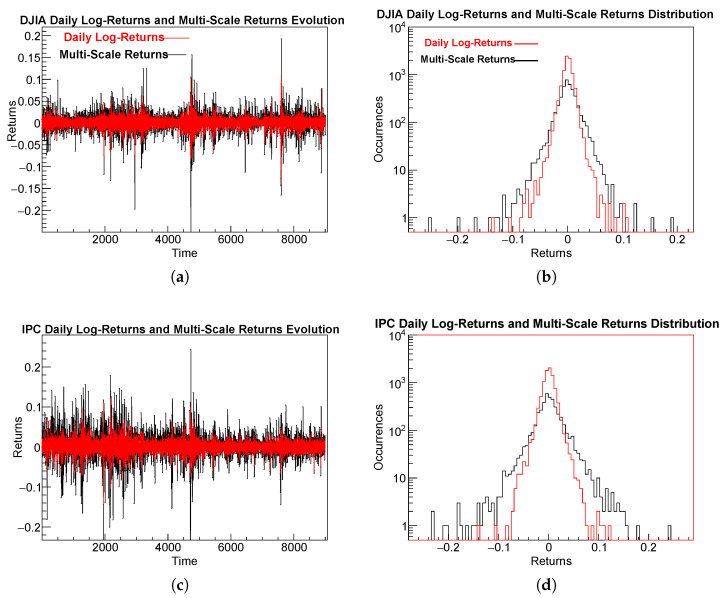
Time evolution of usual daily log-returns (red line) and TReturns (darker line) for the DJIA (**a**) and IPC (**c**). Panels (**b**) DJIA and (**d**) IPC compare the empirical distributions of multi-scale returns (darker line) and usual daily log-returns (red line).

From the definition of TReturns given in Equation (2), it is easy to see that TReturns are, on average, larger in magnitude than usual daily returns. This can be observed in panels of Figure 4a–d. Panels (a,c) show the evolution of TReturns series alongside the usual daily log-returns for the DJIA and IPC, respectively. Panels (b,d) compare the empirical distributions of multi-scale with those of usual daily returns for the DJIA and IPC, respectively.

As is also seen in Figure 4, the variance of multi-scale returns is consistently larger than that of daily returns. This is natural: TReturns are computed over time windows that typically exceed a single day, depending on the durations of uninterrupted trends, and their magnitudes therefore exceed those of usual daily log-returns.

Viewed from another perspective, because multi-scale returns are logarithmic quantities accumulated over several consecutive days during which prices either rise or fall, each TReturn is effectively the sum of multiple same-signed daily log-returns. It follows directly that multi-scale returns are, in general, larger in magnitude than standard one-day returns.

Table 5 summarizes the main descriptive statistics for the daily log-returns and multi-scale return time series of the DJIA and IPC over the period from  2 January 1990 to  17 October 2025.

The four samples show statistical properties typical of financial return distributions. All means are close to zero, indicating the absence of significant drift at both daily and multi-scale returns. At the multi-scale level, standard deviations are roughly twice those of their daily counterparts $\sigma_{\mathrm{multi}}/\sigma_{\mathrm{daily}}\approx 2$, as a consequence of the cumulative nature of trend-based aggregation.

Both indices display mild negative skewness, indicating a slightly higher probability of large negative fluctuations. The DJIA exhibits stronger negative skewness, particularly in the multi-scale case (≈−0.70), suggesting a greater asymmetry in downward movements.

All kurtosis values are substantially larger than 3, confirming leptokurtic behavior with heavy tails. The DJIA exhibits the heaviest tails (kurtosis ≈12), while the IPC values range between 6 and 7, remaining far from Gaussianity but somewhat less extreme. Even accounting for the increased duration and amplified volatility inherent to multi-scale trend returns, these results are consistent with well-known stylized facts of financial return distributions.

### 4.5. TReturns Distribution: Low and Medium Variations

Once it is confirmed that the durations of uninterrupted trends decay according to an exponential law, we observe that the distribution of TReturns also exhibits an exponential decay in the low and intermediate value regions, on both the positive and negative sides of the distribution. The fitting range for the exponential regime was selected by visually identifying the upper bound of the central region, where the exponential behavior is most clearly and robustly observed for both the positive and negative branches.

**Figure 5:**
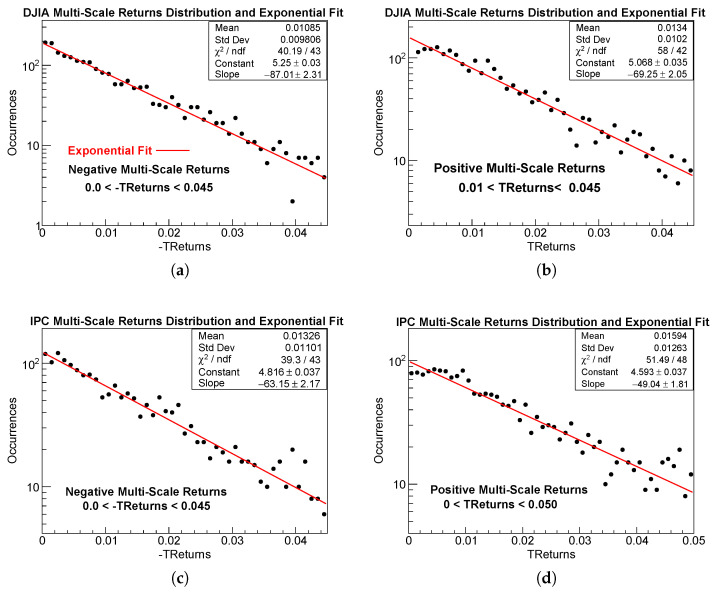
Exponential fits (red lines) to the low and medium value regions of the TReturns distributions (dark bullets), plotted on a log-linear scale. Panels (**a**,**b**) correspond to the DJIA negative and positive TReturns, respectively, while panels (**c**,**d**) show the IPC negative and positive TReturns. Negative values are represented using -TReturns. The fitted regions are indicated both graphically and numerically in each panel and are also reported in Table 6. In all cases, the exponential model provides an adequate description of the data over the fitted ranges.

The result of this approach is illustrated in Figure 5, where the positive and negative regions of the TReturn distributions for the four samples are shown on a log-linear scale, together with their corresponding exponential fits.

For ease of comparison, and because logarithmic quantities cannot be negative when plotted on a log-linear scale, instead of fitting negative returns directly, we fit the low and medium regions of their additive inverse, -TReturns. The resulting exponential fit parameters for the model defined in Equation (3) are reported in Table 6.

As explained in Section 4.2, Table 6 reports the original ROOT fit parameters, where *b* denotes the Slope of the exponential model. A subscript *L* or *R* is used to denote the parameters corresponding to the left (negative) or right (positive) sides of the multi-scale return distribution.

#### Comments on Goodness of the Exponential Fits: TReturns in the Central Region

From Table 6, which reports the exponential fit parameters for the low and medium-value regions of the positive and negative sides of the multi-scale return distributions, it is clear that all four datasets exhibit statistically acceptable exponential fits, with $\chi^{2}/\mathrm{ndf}$ values close to unity. No systematic curvature or residual structure is observed, indicating that the exponential model reliably captures the behavior in these regions reliably.

The decay parameters show clear differences between markets and between daily and multi-scale returns. Multi-scale returns display large slopes, reflecting a faster decay after trend-based aggregation. A mild but consistent asymmetry is also present: for both indices, the negative side decays slightly faster than the positive one ($|Slope_{L}|>|Slope_{R}|$), in agreement with the negative skewness reported earlier. Overall, the exponential regime is robust on both sides of the distribution.

### 4.6. TReturns Distribution: Extreme Variations

In this work, all asymptotic power-law fits were performed using the same procedure implemented in the ROOT framework. We begin with the histogram of multi-scale returns, considering either TReturns for the positive tail or -TReturns for the negative tail, restricted to the asymptotic region selected as follows:

For multi-scale returns, the fitting range $\left(x_{\min},x_{\max}\right)$ of the power-law regime was selected by restricting the analysis to the asymptotic tail region not constrained by the exponential central regime, which required typically the exclusion of one or two extreme outliers. On the other hand, for usual daily returns, the power-law fitting range was selected by visually identifying the asymptotic tail region and refined through simple trial-and-error tests to ensure a stable linear behavior in log–log scale. This procedure for selecting the power-law fitting range is standard practice in econophysics and related empirical studies.

From histogram to TGraph.

A TGraph in ROOT is an unbinned two-column dataset that stores pairs $\left(x_{i},y_{i}\right)$ as individual points. Converting a histogram into a TGraph allows us to fit directly in log–log space, eliminate empty bins, and avoid bin-induced distortions. In our context,

$$x_{i}=\mathrm{TReturns},\;N\left(x_{i}\right)=\mathrm{counts}\;\mathrm{in}\;\mathrm{bin}\;i,$$

and the graph stores the pairs

$$\left(\log_{10}x_{i},\;\log_{10}N\left(x_{i}\right)\right)$$

for all non-empty bins.

Power-law fit.

Once the TGraph is constructed, we perform a least-squares linear fit to the model:

(7) $$\log_{10}N\left(x\right)=p_{0}+p_{1}\;\log_{10}x,$$

where $p_{0}$ and $p_{1}$ appear in the ROOT output as Constant and Slope, respectively. This corresponds to a power-law probability density function (PDF):

$$N\left(x\right)\propto x^{\;p_{1}},$$

so that the standard tail exponent is

(8) $$\alpha =-p_{1}.$$

**Figure 6:**
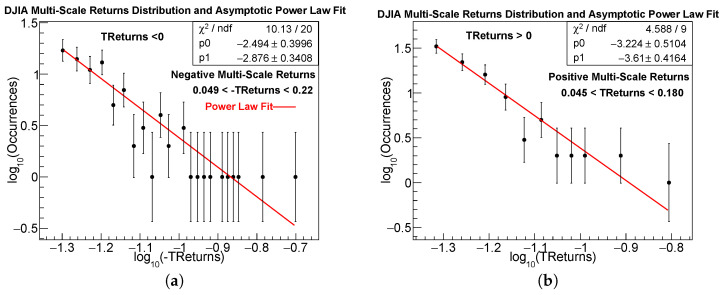

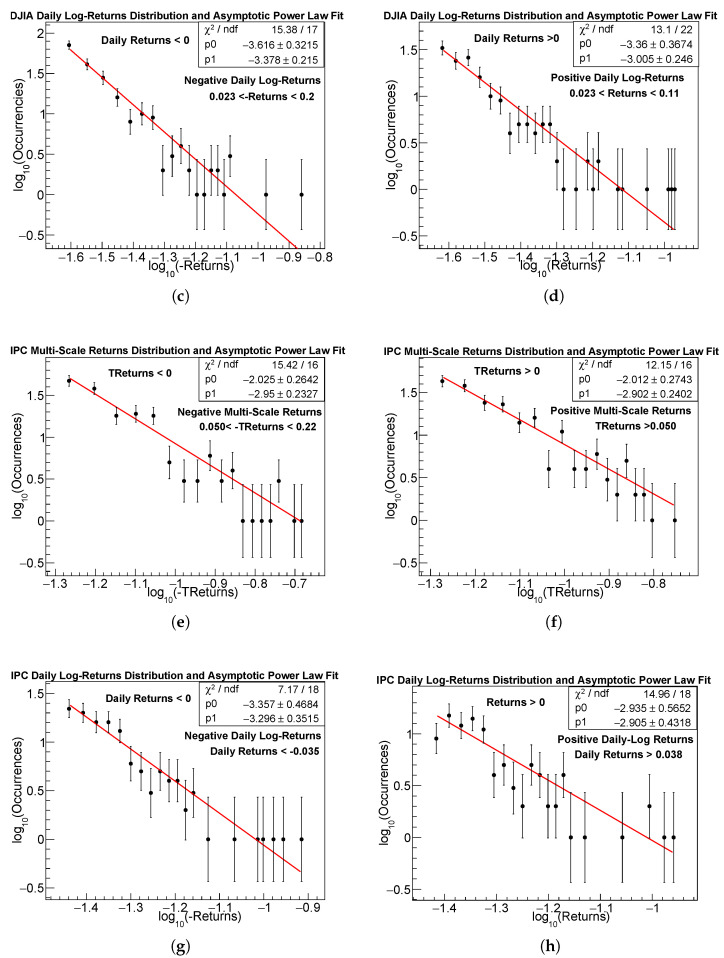
Power-law fits (red lines) of the asymptotic positive and negative tails of the TReturns and the usual daily log-return distributions for the DJIA and IPC, plotted on a log–log scale. Negative tails are represented using -TReturns for multi-scale returns, with an analogous treatment for daily returns. Panels (**a**,**b**) show the negative and positive asymptotic tails of DJIA multi-scale returns, respectively, while panels (**c**,**d**) correspond to the negative and positive asymptotic tails of IPC multi-scale returns. Panels (**e**,**f**) display the negative and positive asymptotic tails of DJIA daily log-returns, respectively, and panels (**g**,**h**) show the corresponding tails for IPC daily log-returns. In all cases, the power-law model provides an adequate description of the extreme-value distributions (dark bullets). The fitted asymptotic regions are indicated both graphically and numerically in each panel, and the corresponding fit parameters are also reported in Table 7.

The fitting procedure described above was applied across all eight cases: positive and negative tails for both The DJIA and IPC, and for both TReturns and usual daily log-returns. Figure 6 displays the power-law fits for extreme positive and negative events in the TReturns (and -TReturns) distributions, together with the corresponding fits for usual daily returns. The results show that, in the asymptotic region selected as described above, the TReturns distributions of both indices follow a power-law decay.

The fit parameters and the regions over which the power-law fits were performed are reported in Table 7.

#### Goodness of Fit and Comparison of Power-Law Exponents

For each of the eight asymptotic regions considered (DJIA and IPC, positive and negative tails, for both TReturns and daily log-returns), ROOT reports the value of $\chi^{2}/\mathrm{ndf}$ for the linear model of Equation (7) applied to the corresponding TGraph. In all cases, the resulting $\chi^{2}/\mathrm{ndf}$ values are moderate and consistent with a stable linear trend within the selected log–log domain. No systematic curvature, clustering, or drift from linearity is observed, indicating that the chosen fitting window $\left(x_{\min},x_{\max}\right)$ successfully isolates the scaling regime.

A comparison among the inferred exponents $\alpha =-p_{1}$ leads to some noteworthy observations:

- Both the DJIA and IPC exhibit tail exponents in the range α≈2.9 to 3.6 for positive and negative TReturns, and α≈2.9 to 3.3 for usual daily returns. Across all four categories (DJIA daily, DJIA TReturns, IPC daily, and IPC TReturns), the positive and negative tail exponents are statistically compatible within uncertainties, showing no evidence of persistent asymmetry in the asymptotic regime.
- Within error bars, the asymptotic exponents of both kind of returns fall within the familiar “inverse-cubic” range α≈3.0 to 3.4 [22,23], in agreement with well-established scaling laws of financial time series.
- Although the estimated tail exponents of TReturns are in some cases marginally smaller than those of daily returns, the theoretical tail index α is not expected to differ from that of usual daily returns. TReturns are finite sums, typically involving only a few consecutive daily log-returns, and sums of a finite number of heavy-tailed variables preserve the same asymptotic power-law exponent α. The slight empirical reduction observed in some cases can be attributed to finite-sample effects and to the amplification of large fluctuations through aggregation, while the underlying tail behavior remains within the inverse-cubic universality class. See the discussion in Section 7.

In summary, the combined evidence from the fitted slopes, their uncertainties, and the $\chi^{2}/\mathrm{ndf}$ diagnostics indicates that (a) all eight empirical tails follow a clear power-law decay in their asymptotic domains, (b) multi-scale returns preserve and enhance the heavy-tailed nature of financial fluctuations, and (c) both markets (DJIA and IPC) exhibit consistent scaling behavior across all return definitions.

Finally, we note that—given the aggregational and multi-scale nature of TReturns—the fitting procedure adopted here was intentionally simple and aimed at discriminating between exponential and power-law regimes. We explicitly acknowledge that least-squares fitting in log–log space may be sensitive to binning choices, noise, cutoff selection, and that no optimal rule exists for selecting the number of bins in histogram construction [24]. More detailed analyses of extreme events, precise parameter estimation, and outliers characterization using alternative, non binning dependent methods, such as that proposed in [25], are left for future work.

### 4.7. Weak Stationarity of Multi-Scale Returns

Although multi-scale returns exhibit a slowly decaying autocorrelation function (ACF) due to their deterministic sign alternating construction, formal weak stationarity tests, such as the augmented Dickey–Fuller and Phillips–Perron tests, consistently reject the presence of unit roots and confirm the stability of their mean and variance. Therefore, despite displaying a weaker form of stationarity than fixed-scale returns, TReturns can be treated as stationary for statistical and econometric purposes, in the same way that conventional financial returns are routinely regarded as stationary despite exhibiting conditional heteroskedasticity. For a more detailed discussion of the stationarity properties of TReturns, the reader is referred to our previous work [17].

## 5. Entropy Analysis

We emphasize that the entropy measures employed in this work are well-established tools in information theory and time-series analysis. Shannon entropy quantifies the dispersion of amplitudes and is closely related to volatility, while permutation entropy captures temporal ordering and directional persistence. The novelty of the present analysis does not lie in these measures themselves, but in their application to multi-scale returns constructed from uninterrupted price trends, and in the informational interpretation of the changes induced by trend-based coarse-graining.

The coarse-graining from daily returns to multi-scale returns (TReturns) can be interpreted as an informational procedure: a temporal aggregation that filters out short-scale (high frequency) fluctuations while preserving the essential directional structure of price dynamics. Each multi-scale return corresponds to an uninterrupted monotonic price trend, either upward or downward, whose magnitude captures the net price change over the entire trend. From a trader’s perspective, the absolute value of a TReturn can be interpreted as the return that an idealized agent would accumulate over that trend [26].

To quantify the reorganization of information induced by the coarse graining operation, we evaluate three complementary measures. The first two are entropy based: Shannon entropy [27], which captures distributional dispersion, and permutation entropy [28], which quantifies temporal disorder. The third is an operational measure of algorithmic complexity [29] based on lossless compression, reflecting the reducibility and structural regularity of each time series. Importantly, none of the series is standardized, since the magnitude of fluctuations itself constitutes a meaningful component of the information generated by the coarse graining process.

### 5.1. Shannon Entropy (Amplitude Dispersion)

In this subsection, Shannon entropy is computed from the empirical distribution of the absolute values of returns (both daily returns and TReturns), in order to quantify amplitude dispersion independently of the return sign. Using absolute returns allows Shannon entropy to probe the spread of fluctuation magnitudes while filtering out directional information, making it particularly suitable for assessing the amplitude effects induced by trend-based coarse-graining.

Shannon entropy should not be interpreted here as a mere alternative volatility proxy, but rather as a quantitative measure of amplitude spreading generated by trend-based aggregation across endogenous time scales, rather than as a measure of randomness or unpredictability. This interpretation is consistent with previous studies that relate entropy measures to volatility and information content in financial time series (e.g., [30,31]), while emphasizing the additional effects induced here by trend-based coarse-graining.

Shannon entropy *H* was computed using K=100 fixed linear bins. The results are summarized in Table 8.

Regarding entropy normalization, we note that $H_{\max}=\log_{2}K$ corresponds to the uniform distribution over the *K* fixed bins. The ratio $H/H_{\max}$ thus measures the proximity of the empirical distribution to the uniform case, providing a natural scale for comparing amplitude dispersion across datasets. All datasets share the same $H_{\max}$ because Shannon entropy was computed using the same fixed number of bins (K=100) in all cases, ensuring that differences in *H* reflect genuine changes in distributional dispersion rather than artifacts of binning or histogram resolution.

Accordingly, Shannon entropy increases markedly for the multi-scale return series. This should not be interpreted as increased randomness, but as increased amplitude dispersion: aggregating uninterrupted trends produces multi-scale returns that extend over a broader numerical range. Shannon entropy thus serves here as a quantitative measure of amplitude spreading induced by trend-based coarse-graining.

Robustness of Shannon entropy.

The stability of the Shannon entropy estimates was assessed by varying the number of bins over K=80,90,100,110, and 120, using a fixed range [0,max|TReturns|] for each financial index. The maximum relative variation with respect to the reference case K=100 is approximately 10.7% and 8.1% for daily returns and multi-scale returns of the DJIA, respectively, and 9.4% and 7.1% for the corresponding IPC series. In all cases, the entropy values vary smoothly with *K* and the qualitative ordering remains unchanged, confirming the robustness of the results.

### 5.2. Permutation Entropy (Temporal Structure)

While Shannon entropy is computed on absolute returns to quantify amplitude dispersion, permutation entropy is evaluated on the original signed return series and is therefore sensitive to temporal ordering and directional structure.

Permutation entropy was computed using embedding dimension m=5 and embedding delay τ=1. The parameter *m* specifies the length of each ordinal pattern and determines how many consecutive points of the time series are compared. In this work, m=5 means that each pattern is constructed from five consecutive values, yielding m!=120 possible ordinal configurations. The parameter τ sets the temporal spacing between the elements of each pattern. Using τ=1 implies that the five values in each pattern correspond to consecutive observations of the series, allowing us to probe short-range temporal structure with maximum resolution.

Permutation entropy quantifies temporal disorder through ordinal patterns. After normalization by $\log_{2}\left(m!\right)$, values close to unity indicate behavior consistent with randomness. Table 9 shows the values of this measure for all return types analyzed in this work.

Permutation entropy drops by approximately one third for TReturns, indicating a strong reduction in temporal randomness. This behavior reflects the effect of trend-based coarse-graining, which suppresses micro-scale noise and reveals persistent, ordered patterns associated with uninterrupted trends. Taken together with the increase in Shannon entropy, these results show that multi-scale returns exhibit greater amplitude dispersion but lower temporal entropy, pointing to a concentration of information across time scales defined by run durations. Since permutation entropy is sensitive to ordinal patterns and directional persistence, its reduction indicates a higher degree of temporal organization in TReturns than in daily returns. While this behavior is consistent with the presence of trend persistence and with deviations from weak-form market efficiency at the coarse-grained level, a detailed investigation of market efficiency lies beyond the scope of the present work and is left for future research.

### 5.3. Compression Analysis (Computational Complexity)

Using the standard Ubuntu zip utility (based on the DEFLATE algorithm), we compressed the plain-text files corresponding to both the daily-return and TReturns series. Data compression provides a practical, operational estimate of Kolmogorov complexity, understood as the length of the shortest description of a dataset [29,32]. More regular or redundant sequences admit shorter descriptions, whereas noise-dominated sequences do not.

Table 10 reveals a clear pattern: files containing TReturns are roughly 50% smaller than their daily-return counterparts, reflecting the intrinsic data reduction produced by trend-based coarse-graining. After ZIP compression, TReturns files remain about half the size of the daily-return files (19 kB vs. 36 kB for the DJIA, and 17 kB vs. 36 kB for the IPC). All datasets compress to approximately 36–38% of their original size, indicating that all series reach the practical compression limit of the DEFLATE algorithm; within this limit, the coarse-grained series remains the most compact.

From an information-theoretic viewpoint, the enhanced compressibility of multi-scale returns is fully consistent with the entropy based results presented above.

It is worth emphasizing that the absolute compression ratios reported in this section depend on the specific compression algorithm employed and should not be interpreted as universal measures of complexity. The relevant result of the compression analysis is the relative comparison between daily returns and multi-scale returns, which should be robust across compression methods, as it reflects structural differences induced by the trend-based coarse-graining rather than algorithm-specific features.

Taken together, the three informational measures considered here indicate that coarse-graining reduces the effective number of degrees of freedom, suppresses microscopic fluctuations, and lowers computational complexity. As a result, multi-scale returns provide a cleaner and more structured representation of market dynamics.

## 6. Discussion

In this section, we discuss the statistical behavior of TReturns and the entropy patterns associated with their construction, highlighting how the coarse-graining procedure shapes both their distributional properties and informational content.

### 6.1. On the Exponential and Power-Law Decay Regimes of TReturns

The empirical distribution of multi-scale returns follows an exponential decay in the central region and a power-law decay in the tails. A plausible explanation for this empirical pattern, consistent with the aggregation of daily log-returns, is that short and medium length trends are the most frequent. Their accumulated log-returns therefore remain relatively small on average, allowing the exponential law governing run durations to propagate naturally into the low and intermediate-value regions of the TReturn distribution.

This mechanism, however, cannot suppress the rarer and larger extreme events, whether arising from exceptionally long trends or from large daily fluctuations. Such events dominate the tail behavior and exclude asymptotic exponential decay, giving rise instead to the observed power-law regime. The exponential decay at low and intermediate return magnitudes and the power-law tails thus appear to originate from distinct dynamical scales: typical runs shaping the central region, and atypical trends shaping the extremes of the multi-scale return distribution.

We emphasize that exponential run-length statistics are well known for idealized random-walk models. However, the emergence of an exponential central region in the TReturns distribution reported here is an empirical result arising from trend-based coarse-graining of real financial data and does not follow trivially from those idealized models. A formal theoretical derivation of this empirical result remains an open problem.

### 6.2. On the Notion of Stationarity of Multi-Scale Returns

No stationarity tests are performed in the present work. The discussion regarding the stationarity properties of multi-scale returns is based on the results reported in our previous dedicated study [17], where the autocorrelation structure and unit-root tests (ADF and Phillips–Perron) were analyzed in detail for the signed TReturns series. These unit-root tests were applied to the signed TReturns series; absolute returns were not considered, as unit-root testing is not designed for magnitude-based processes.

That analysis showed that, despite the deterministic sign alternation and the slow, oscillatory decay of the autocorrelation function induced by the construction of TReturns, standard unit-root tests consistently reject the presence of a unit root. At the same time, multi-scale returns do not satisfy the usual ACF-based criteria associated with conventional weak stationarity. This apparent coexistence reflects a weaker notion of stationarity than the classical one, characterized by stable mean and variance but persistent structural dependence.

Such a weak form of stationarity is sufficient, for practical purposes, to justify treating TReturns as stationary for the distributional, informational, and compression-based analyses carried out in this work.

### 6.3. Information-Theoretic Interpretation

Entropy analyses clarify how trend-based coarse-graining modifies the informational structure of the time series. Shannon entropy increases because aggregation yields TReturns with a broader amplitude range, reflecting greater dispersion rather than increased randomness. In this work, Shannon entropy is evaluated for the absolute values of TReturns and should be interpreted as a measure of amplitude dispersion induced by trend-based coarse-graining, rather than as an indicator of randomness.

While permutation entropy has been widely used in financial time series, its reduction for multi-scale returns indicates that trend aggregation suppresses high-frequency noise and enhances temporal order, rather than reproducing known results for daily returns.

From the perspective of computational complexity, TReturns files compress to approximately half the size of their daily-return counterparts, even when the same lossless DEFLATE algorithm is used. This indicates a marked reduction in algorithmic complexity: removing high-frequency oscillations yields a representation with greater redundancy and more regular structure. Coarse-graining therefore not only alters statistical entropy but also shortens the effective description length of the data.

Taken together, these three measures consistently reveal a coherent information-theoretic signature of multi-scale returns: their magnitude range broadens, temporal randomness decreases, and algorithmic complexity is reduced. In this sense, multi-scale returns provide a compact intermediate-scale representation of market dynamics, capturing the essential signals associated with persistent trends across multiple time scales.

We end this section by noting that related connections between entropy reduction, coarse-graining, and the emergence of deterministic structure in financial time series have been discussed in the context of entropy-corrected stochastic modeling [33]. Here, we adopt a complementary perspective by using entropy measures to characterize the informational effects induced by trend-based aggregation on a newly defined multi-scale observable.

## 7. Conclusions

This study analyzes the statistical characteristics of multi-scale returns (TReturns), constructed by aggregating daily log-returns over uninterrupted upward or downward price trends in the DJIA and IPC indices. Since the durations of these trends are typically exponentially distributed, the resulting TReturns naturally reflect this distribution in their central region.

This robust empirical pattern supports the proposal of a new stylized fact: *coarse-grained returns constructed over exponentially distributed uninterrupted trend durations exhibit an exponential decay in their central region before transitioning to a heavy-tailed regime*. Unlike usual daily returns, for which no universal model exists for the central part of the distribution, TReturns display a clear exponential behavior, supported by empirical evidence. We emphasize that we have constructed a new multi-scale random variable from the complete sequence of daily returns, in direct analogy with other classical stylized results, and that this variable reveals a robust exponential central behavior, which supports its interpretation as a new stylized fact within a multi-scale representation of financial fluctuations.

However, the exponential pattern breaks down in the extreme tails. In these regions, the TReturns distributions follow a power-law behavior, similar to that observed for standard daily log-returns. This occurs because TReturns are finite sums of daily price changes accumulated over randomly varying time intervals; consequently, extreme values are still governed by the same mechanisms that generate heavy tails in financial data. The estimated tail exponents for the DJIA and IPC, ranging from approximately 2.90 to 3.61, are consistent with the well-known inverse-cubic law.

The emergence of an exponential decay in the central region of the TReturns distribution can be understood as follows. Although TReturns are sums of daily log-returns whose distributions are not exponential, the exponential behavior does not originate from the returns themselves, but from the distribution of trend durations. Since the run lengths $m_{i}$ are exponentially distributed, this randomness in the aggregation time scale induces an exponential regime in the low and intermediate return values. In contrast, the asymptotic power-law tails are inherited from the heavy-tailed nature of daily returns. Indeed, it is well established that power-law tails remain stable under the finite summation of heavy-tailed variables [34,35,36].

It is worth emphasizing that the joint emergence of an exponentially distributed central region and asymptotic power-law tails is a recurring phenomenon in many complex systems, as discussed in Section 1.2. Its relevance lies in the coexistence of two distinct dynamical regimes: short-range, memoryless dynamics shaping the central region, and rare, large-scale events governing the asymptotic behavior. The reappearance of this particular combination of distributions in multi-scale financial returns further supports the view that markets share common organizing principles with other complex systems, in which microscopic self-organization produces universal statistical patterns.

In this work, we provide a new example of such a stochastic process within a complex system: multi-scale financial returns. In this case, a combined exponential central region and asymptotic power-law tail distributional structure emerges naturally. We also propose a physically motivated mechanism that can explain both the exponential component and the emergence of the asymptotic power-law behavior at the mesoscopic level of TReturns.

In addition to the distributional properties of TReturns reported in this work, the information analyses yield the following results. Shannon entropy increases because trend aggregation broadens the amplitude range of returns, while permutation entropy decreases sharply, indicating a reduction in temporal randomness and the emergence of order. Compression analysis further reveals a substantial reduction in algorithmic complexity and noise. Taken together, these results show that multi-scale returns retain the essential market information in a more ordered and potentially more analyzable form.

From a practical perspective, it is natural to interpret the absolute value of TReturns as the profit sequence of an idealized trader endowed with complete information (or perfect luck), who enters a long position at the onset of each upward trend, closes it at the trend’s end, and immediately switches to a short position at the beginning of every downward trend [26]. This interpretation highlights the intrinsically multi-scale and structurally induced nature of TReturns. A formal derivation of these empirical results remains an open task for future work.

An additional implication of the trend-based coarse-graining is the existence of a time scale below which price fluctuations are largely dominated by noise and directional information becomes weak or unreliable. Importantly, this threshold is not fixed, but depends on the market, asset, operating conditions, and the trader’s risk aversion, as well as on the adopted trading horizon (e.g., intraday versus multi-day). Transaction costs, commissions, execution delays, and other market frictions further affect the effective noise threshold and would significantly reduce the realizability of short-horizon trading decisions. Consequently, this time scale must be determined empirically by the trader or analyst.

In this sense, TReturns are best understood as a structural reference sequence that filters short-horizon noise and captures the organization of price fluctuations across endogenous time scales, rather than as a prescription for practical trading.

In conclusion, our results suggest that multi-scale returns provide a consistent coarse-grained representation of financial fluctuations. They preserve the exponential signature associated with uninterrupted trend durations, reproduce the heavy-tailed asymptotic behavior characteristic of financial returns, satisfy a practical notion of weak stationarity, and reveal clear information reorganization induced by coarse-graining.

The results presented in this work highlight trend-based coarse-graining as a natural mechanism for uncovering intermediate-scale structure in financial time series.

## Figures and Tables

**Table 1:** Descriptive statistics of run durations for DJIA and IPC, combining both uptrends and downtrends.

Index	Daily Records	Runs	Uptrends	Downtrends	Mean	Std Dev	Skewness	Kurtosis
DJIA	9011	4595	2297	2298	2.020	1.352	2.077	6.251
IPC	8996	4189	2095	2094	2.147	1.513	1.887	4.155

**Table 2:** Exponential fit parameters for overall (combined) trend durations.

Index	Constant *a*	Slope *b*	$\chi^{2}/\mathrm{ndf}$	Notes
DJIA	8.53±0.03	−0.722±0.011	8.925/11	all trends
IPC	8.235±0.027	−0.625±0.010	2.61/9	all trends

**Table 3:** Run statistics for uptrends and downtrends.

Market	Trend	Entries	Mean	Std Dev	Skew	Kur
DJIA	Downtrends	2298	1.830	1.206	1.993	5.800
	Uptrends	2297	2.091	1.487	2.103	5.859
IPC	Downtrends	2094	2.045	1.424	2.014	5.291
	Uptrends	2095	2.249	1.591	1.689	3.209

**Table 4:** Exponential fits parameters for uptrends and downtrends.

Market	Trend	Constant *a*	Slope *b*	$\chi^{2}/\mathrm{ndf}$
DJIA	Downtrends	7.912±0.036	−0.7865±0.0162	7.998/8
	Uptrends	7.701±0.036	−0.6572±0.0138	8.034/11
IPC	Downtrends	7.646±0.038	−0.6738±0.0149	4.145/9
	Uptrends	7.393±0.037	−0.5771±0.0133	1.512/8

**Table 5:** Descriptive statistics for DJIA and IPC daily and multi-scale returns (2 January 1990–17 October 2025).

Dataset (Returns)	N	Mean	Std	Min	Max	Skewness	Kurtosis
DJIA multi-scale returns	4596	0.0006	0.0217	−0.2490	0.1931	−0.697	12.08
DJIA daily returns	9010	0.0003	0.0109	−0.1384	0.1076	−0.371	12.64
IPC multi-scale returns	4191	0.0012	0.0315	−0.2326	0.2442	−0.231	7.22
IPC daily returns	8995	0.0006	0.0138	−0.1431	0.1215	−0.001	6.67

**Table 6:** Exponential fit parameters for the low and medium value regions of the positive and negative sides of the TReturns distribution. The constant parameter *a* is omitted because it does not affect the decay rate; only the slopes are reported.

Market TReturns	${\mathrm{Slope}}_{L}$	Fitting Region	$\chi_{L}^{2}/\mathrm{ndf}$	${\mathrm{Slope}}_{R}$	Fitting Region	$\chi_{R}^{2}/\mathrm{ndf}$
DJIA TReturns	−87.01 ± 2.31	(−0.045,0)	40.19/43	−69.25 ± 2.05	(0.01,0.045)	58.00/42
IPC TReturns	−63.15 ± 2.17	(−0.045,0)	39.3/43	−49.04 ± 1.81	(0.00,0.050)	51.49/48

**Table 7:** Asymptotic power-law fits for multi-scale returns (TReturns) and usual daily log-returns for the DJIA and IPC. The fits correspond to the log–log PDF representations shown in Figure 6. The PDF tail exponent is defined as $\alpha =-p_{1}$.

Market	Return Type	Tail	$p_{0}$	$p_{1}$	α	Fitting Region	$\chi^{2}/\mathrm{ndf}$
DJIA	TReturns	Negative	−2.494±0.400	−2.876±0.341	2.876	(−0.22,−0.049)	10.13/20
		Positive	−3.224±0.510	−3.610±0.416	3.610	(0.045,0.180)	4.588/9
DJIA	Daily log-returns	Negative	−3.616±0.322	−3.378±0.215	3.378	(−0.20,−0.023)	15.38/17
		Positive	−3.360±0.367	−3.005±0.2460	3.005	(0.023,0.11)	13.1/22
IPC	TReturns	Negative	−2.025±0.264	−2.950±0.233	2.950	(−0.22,−0.050)	15.42/16
		Positive	−2.012±0.274	−2.902±0.240	2.902	(0.050,∞)	12.15/16
IPC	Daily log-returns	Negative	−3.357±0.469	−3.296±0.352	3.296	(−∞,0.035)	7.17/18
		Positive	−2.935±0.565	−2.905±0.432	2.905	(0.038,∞)	14.96/18

**Table 8:** Shannon entropy and redundancy computed for absolute daily log-returns and absolute multi-scale returns using a fixed number of bins (K=100).

Dataset	Total	Non-Empty Bins	*H* [bits]	$H_{\max}$ [bits]	$H/H_{\max}$	Redundancy
DJIA absolute daily log-returns	9010	38	2.996	6.644	0.451	0.549
DJIA absolute TReturns	4596	55	3.941	6.644	0.593	0.407
IPC absolute daily log-returns	8995	38	3.391	6.644	0.510	0.490
IPC absolute TReturns	4191	66	4.461	6.644	0.671	0.329

**Table 9:** Permutation entropy (m=5, τ=1) for DJIA and IPC daily and multi-scale returns.

Dataset	$H_{\mathrm{perm}}$ [bits]	$H_{\mathrm{perm}}/\log_{2}\left(m!\right)$
DJIA daily returns	6.882	0.996
DJIA TReturns	4.574	0.662
IPC daily returns	6.884	0.997
IPC TReturns	4.576	0.663

**Table 10:** File sizes (in kilobytes) for daily returns and TReturns before and after ZIP compression, together with the percentage of the original size that remains after compression.

Dataset	Plain Text (kB)	ZIP (kB)	Compression (%)
DJIA daily returns	99	36	36%
DJIA TReturns	50	19	38%
IPC daily returns	98	36	37%
IPC TReturns	45	17	38%

## Data Availability

The datasets generated and/or analyzed during the current study are publicly available in the Zenodo repository at https://doi.org/10.5281/zenodo.17781871.

## Abbreviations

The following abbreviations are used in this manuscript:

DJIA	Dow Jones Industrial Average
IPC	Índice de Precios y Cotizaciones (Mexican Stock Exchange Index)
PDF	Probability Density Function
ACF	Auto-Correlation Function
